# Association between Metabolic Syndrome Status and Daily Physical Activity Measured by a Wearable Device in Japanese Office Workers

**DOI:** 10.3390/ijerph20054315

**Published:** 2023-02-28

**Authors:** Yukako Yamaga, Thomas Svensson, Ung-il Chung, Akiko Kishi Svensson

**Affiliations:** 1 Precision Health, Department of Bioengineering, Graduate School of Engineering, The University of Tokyo, Tokyo 113-8656, Japan; 2 Graduate School of Health Innovation, Kanagawa University of Human Services, Kawasaki 210-0821, Japan; 3 Department of Clinical Sciences, Lund University, Skåne University Hospital, CRC, Jan Waldenströms Gata 35, 205 02 Malmö, Sweden; 4 Clinical Biotechnology, Center for Disease Biology and Integrative Medicine, Graduate School of Medicine, The University of Tokyo, Tokyo 113-8656, Japan; 5 Department of Diabetes and Metabolic Diseases, The University of Tokyo, Tokyo 113-0033, Japan

**Keywords:** active minutes, metabolic syndrome, physical activity, step count, wearable device

## Abstract

(1) Background: This study examined the cross-sectional association between metabolic syndrome (MetS) status classified into three groups and daily physical activity (PA; step count and active minutes) using a wearable device in Japanese office workers. (2) Methods: This secondary analysis used data from 179 participants in the intervention group of a randomized controlled trial for 3 months. Individuals who had received an annual health check-up and had MetS or were at a high risk of MetS based on Japanese guidelines were asked to use a wearable device and answer questionnaires regarding their daily life for the entire study period. Multilevel mixed-effects logistic regression models adjusted for covariates associated with MetS and PA were used to estimate associations. A sensitivity analysis investigated the associations between MetS status and PA level according to the day of the week. (3) Results: Compared to those with no MetS, those with MetS were not significantly associated with PA, while those with pre-MetS were inversely associated with PA [step count Model 3: OR = 0.60; 95% CI: 0.36, 0.99; active minutes Model 3: OR = 0.62; 95% CI: 0.40, 0.96]. In the sensitivity analysis, day of the week was an effect modifier for both PA (*p* < 0.001). (4) Conclusions: Compared to those with no MetS, those with pre-MetS, but not MetS, showed significantly lower odds of reaching their daily recommended PA level. Our findings suggest that the day of the week could be a modifier for the association between MetS and PA. Further research with longer study periods and larger sample sizes are needed to confirm our results.

## 1. Introduction

Metabolic syndrome (MetS) is a global epidemic and growing public health concern. Although the definition of and criteria for MetS vary among health organizations in different countries, it is a major risk factor contributing to cardiovascular disease and type 2 diabetes mellitus [1], and leads to higher mortality [1,2]. While the biological and pathophysiological mechanisms of MetS remain an active area of research, it is clearly a cluster of health conditions that include obesity, dyslipidemia, hypertension, hyperglycemia, and insulin resistance, which are intricately associated with lifestyle factors such as lower physical activity (PA) [3,4,5], imbalanced nutrition [6], and genetics and epigenetics [6,7]. While estimations of its prevalence vary, more than one quarter of the world’s population, or approximately one billion people, are estimated to have MetS [7]. Therefore, public education, the awareness of health hazards caused by MetS, and appropriate measures to assess MetS are expected to limit the impact on society [7]. 

Recent studies have suggested that PA may play an important role in improving health and preventing MetS. In fact, greater levels and duration of PA are linked to a significant reduction in MetS prevalence [5]. In 2020, the World Health Organization (WHO) updated their guidelines on PA and sedentary behavior for different population groups to enhance health outcomes [8]. In Japan, the Ministry of Health, Labour and Welfare of Japan (MHLW) has issued the Exercise and Physical Activity Reference for Health Promotion (EPAR) 2013, which is a revision of the 2006 guidelines [9]. However, according to the 2019 Japanese National Health and Nutrition Survey (NHNS), the average step count of those aged 20–64 years old was below the target value [10]. Although studies that have examined the effect of PA interventions have shown improvements in hyperlipidemia [11], hypertension [12] and diabetes mellitus [13], much of the evidence on the beneficial effects of PA on metabolic risk in population-based studies have been obtained using self-reported questionnaires [3,4], which are limited by potential recall bias.

Consumer wearable devices have risen in popularity over the past several years, as they allow individuals to monitor various health parameters, such as PA [14], sleep duration [15], heart rate [16], and energy consumption. The availability and use of wearable devices is expected to increase, as they can encourage better health behaviors and help individuals manage their health through daily monitoring [14]. Additionally, as consumer wearable devices can capture various PA-related parameters, they are also useful for researchers in gathering data in free-living conditions. This can enhance investigations into multiple conditions regarding PA level, although challenges related to the reliability and validity of different devices and models remain an issue [17,18].

Given this background, the aim of this study was to examine the association between MetS and objectively measured PA levels. We studied this by: (1) measuring PA levels using a wearable device and comparing them with PA reference values in Japan, and determining MetS status based on data derived from annual health checkup (AHC) data and MetS criteria in Japan [19]; and (2) examining the potential effect modification of the day of the week (weekday or weekend/holiday) in sensitivity analysis. We hypothesized that MetS and pre-MetS would be inversely associated with objectively measured PA-level.

## 2. Materials and Methods

### 2.1. Study Design

Participants were full-time professionals, managerial, or clerical workers recruited from five companies, each with more than 1000 employees located in the Tokyo metropolitan area in Japan. The original study was a randomized controlled trial (RCT) of a smartphone lifestyle intervention application. Participants eligible for the RCT were employees that had completed the annual health check-up (AHC) and had been categorized with MetS or were considered to be at risk of MetS based on their AHC results. The participants of the original RCT were recruited among 7437 eligible employees. A total of 272 participants were enrolled and randomized into 2 arms: 181 were in the intervention group and 91 were in the control group. Participants in the intervention group were asked to use a wearable device 24 h per day and a dedicated smartphone application for the entire 3 months of the study. Participants in the control group completed a questionnaire using a dedicated smartphone application. All participants received detailed information about the study both in writing and face-to-face. Information provided to study participants included, but was not limited to, ethical considerations, the purpose of the study, and the right to discontinue at any time without penalty. All participants understood that participation was entirely voluntary and provided written informed consent. The study was approved by the Ethics Committee of the School of Engineering, the University of Tokyo (approval number: KE18-44). Participants in the intervention group were gifted the wearable device as an incentive if they completed the final questionnaire at the end of the study. Participants in the control group were gifted the wearable device at the end of the study.

### 2.2. Data Collection

#### 2.2.1. Wearable Device

The Fitbit Versa (FV), a consumer wearable device manufactured by Fitbit Inc. (San Francisco, CA, USA, https://www.fitbit.com (accessed on 19 April 2022), was used in this study. The FV connects to a dedicated smartphone application using Bluetooth technology and is designed to measure daily information such as step count, duration of PA, heart rate, sleep time, distance, and consumed calories. These data are automatically uploaded to the application when the device is synchronized to the smartphone application via Bluetooth. In this study, participants were instructed to wear the FV for 24 h per day except when the device was charging or they were bathing. Recent studies evaluating the accuracy of consumer wearable devices compared with research-grade devices indicated that Fitbit was examined the most frequently [17] and had sufficient accuracy to measure step count [20] and energy expenditure showing the equivalence zone [21].

#### 2.2.2. Questionnaires

Participants in both arms of the study were asked to complete questionnaires through a smartphone application at the start and end of the study. The intervention arm additionally answered daily questions every morning and afternoon during the study period. Questions originally established and asked at the start and end of the study aimed to assess participants’ lifestyle, psychological and physical condition, medical history, family history, socio-economic status, smoking, alcohol, diet, exercise, sleep status, working condition, stress, and health awareness. Daily questions were related to subjective sleep assessment, alcohol consumption, smoking, diet, exercise, and stress level, which are reflective of each participant’s behavior and health condition on the corresponding night (for sleep) and day (for the remaining lifestyle factors).

### 2.3. Study Population

**Figure 1:**
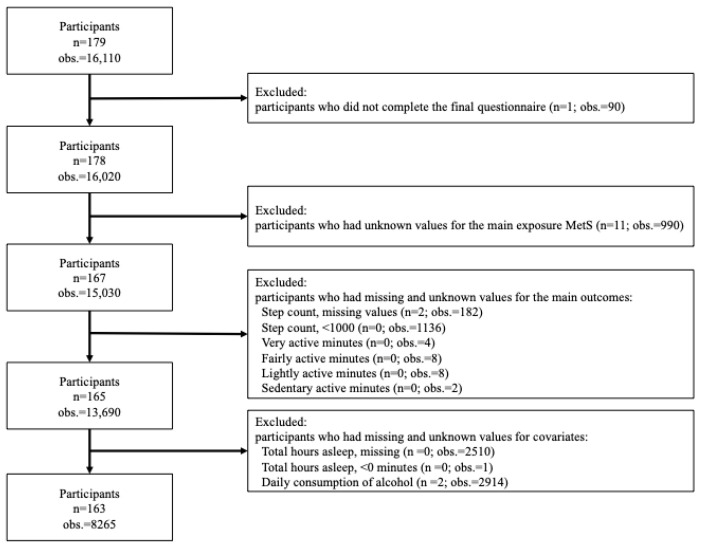
Flowchart detailing the inclusion and exclusion of participants and observations in the present study. Abbreviations: obs: observations.

We conducted a secondary analysis of data from the intervention group of a 90-day randomized controlled trial conducted between 3 December 2018 and 2 March 2019. As the present study aimed to examine the association of MetS prevalence with free-living PA in Japanese office workers, only the intervention group (179 participants with 16,110 observations) was eligible for analysis (Figure 1). Two participants originally randomized to the intervention group declined to participate. Due to the characteristics of the dataset, we excluded any participants who did not complete the final questionnaire (*n* = 1; 90 observations) and participants whose exposure (MetS) status could not be classified (*n* = 11; 990 observations) due to missing information on waist circumference (WC). Participants and observations were further excluded if they had missing values on daily step count (*n* = 2; 182 observations), unreasonable values on daily step count (daily step count < 1000: *n* = 0; 1136 observations), and missing values on very active minutes (*n* = 0; 4 observations), fairly active minutes (*n* = 0; 8 observations), lightly active minutes (*n* = 0; 8 observations), and sedentary minutes (*n* = 0; 2 observations). Lastly, we excluded those with missing values on total sleep time (TST; *n* = 0; 2510 observations), unreasonable values on TST (0 min: *n* = 0; 1 observation), and missing values on daily alcohol consumption (*n* = 2; 2914 observations). As a result, 163 participants with 8265 observations were included in the present analysis.

### 2.4. Metabolic Syndrome Status (Main Exposure)

Participants were classified into three categories of MetS (no-MetS, pre-MetS, and MetS) using AHC data according to the criteria outlined by the Japanese Society of Internal Medicine in 2005 [19]. The prevalence of MetS was determined using the obligatory criterion for WC (≥85 cm in men and ≥90 cm in women) in addition to at least 2 non-obligatory criteria: triglycerides (TG) ≥ 150 mg/dL and/or high-density lipoprotein cholesterol (HDL-C) < 40 mg/dL; systolic blood pressure (SBP) ≥ 130 mmHg and/or diastolic blood pressure (DBP) ≥85 mmHg; or fasting blood glucose (FBG) ≥ 110 mg/dL. Participants who met the obligatory criterion for WC but met only 1 non-obligatory criterion were categorized as having pre-MetS. On the other hand, participants who did not meet the obligatory criterion for WC were categorized as no-MetS [22].

### 2.5. Physical Activity (Main Outcome)

We used 2 main outcome measures, daily step count and active minutes, to represent participants’ PA. Both outcome measures were obtained from the wearable device and were used as continuous outcome variables in the main analyses. For step count, we excluded any missing values (*n* = 2; 182 observations) and any daily observations with fewer than 1000 steps (*n* = 0; 1136 observations), as such low daily counts were considered improbable and would most likely represent inadequate use of the wearable device.

As reference values for daily step count are suggested in the EPAR (9000 steps in men and 8500 steps in women aged 18–64 years old), we established a binary variable based on these cutoffs (0: did not achieve the reference daily steps, 1: achieved the reference daily steps).

The EPAR also suggests a reference PA intensity as 60 min of moderate-to-vigorous intensity physical activity (MVPA) based on the recommended minimum PA per day for people aged 18–64 years old (23 metabolic equivalents (METs) h/week) [9]. Activity measures obtained from the FV were classified into four PA intensity categories [23,24]: (1) sedentary active minutes (<1.5 METs), (2) lightly active minutes (1.5–3.0 METs), (3) fairly active minutes (3.0–6.0 METs), and (4) very active minutes (>6.0 METs). As fairly active minutes and very active minutes are likely comparable to the MVPA recommended by the EPAR (equivalent to >3 METs), we combined these categories into a single variable called active minutes [24]. We additionally established a binary variable based on the cutoff for active minutes (0: did not achieve the reference PA intensity (<3 METs h/day), 1: achieved the reference PA intensity (≥3 METs h/day)).

### 2.6. Covariates

Covariates were determined based on their known or suspected association with the exposure and outcomes. Covariates were related to participants’ demographic information, lifestyle, socio-economic situation, and general health awareness, and were obtained from four main sources: (1) the study’s baseline questionnaire, (2) daily questions, (3) annual health check-up data, and (4) the wearable device. Demographic information included sex and age (continuous); lifestyle factors included eating habits with self-reported balanced food intake (yes/no), daily alcohol consumption (ethanol intake <20 g or ≥20 g as per the MHLW’s recommended daily limit [25]); TST (continuous, hours); smoking status (non-smoker, past smoker, current smoker <20 cigarettes per day, or current smoker ≥20 cigarettes per day); self-reported hours of overtime work in the month preceding the start of the study (continuous, hours); and living arrangement (living alone/living with someone). Socio-economic status was categorized according to self-reported annual income (<10 million JPY or ≥11 million JPY). We constructed a variable to represent heath awareness using five self-reported items from the study’s baseline questionnaire to obtain an overall composite score. The items were: (1) “How often (per week) do you exercise (physically demanding sports/physical activity) for at least 30 min?” (0: no exercise, 1: 1–2 days, 2: ≥3 days), (2) “How often do you walk or do equivalent physical activity for an hour or more?” (0: never, 1: <2 times, 2: ≥3 times per week), (3) “Do you consciously take opportunities to move your body, such as taking the stairs instead of the elevator?” (0: no, 1: yes), (4) “Do you regularly measure your weight?” (0: no, 1: yes), and (5) “Do you have a dental check-up at least once a year?” (0: no, 1: yes). The total score of the composite variable ranged between 0–7 points, with higher scores indicating greater health awareness. Finally, we used the item, “How do you feel about your current health status?” to represent participants’ self-rated health status (SRH), which ranged from 0–5 points (very poor, poor, fair, good, or very good). To determine the day of the week during the study, we created a binary variable (weekdays or weekends/holidays) to classify days according to the Japanese calendar.

### 2.7. Statistical Analysis

Baseline characteristics according to MetS classification were compared using the chi-squared test for categorical variables, the Kruskal–Wallis test for continuous variables and repeated-measures variables (i.e., variables collected daily using the daily questionnaire: daily alcohol intake), or via the wearable device (step count, active minutes, and TST).

Associations between MetS status and PA level (step count and active minutes) based on the EPAR reference values were estimated using multilevel mixed-effects logistic regression models by considering 95% confidence intervals (CI) while accounting for goodness of fit. Model 1 included age and sex as fixed effects. Model 2 additionally included the day of the week (weekday or weekend/holiday), and eating habit, smoking, overtime hours, living arrangement, socio-economic status (all fixed effects), alcohol consumption and TST (random effects) as lifestyle factors. Moreover, because participants’ PA may be influenced by health awareness and daily behaviors, we adjusted for health awareness and SRH [26] as fixed effects in Model 3. All mixed models were analyzed using the unstructured covariance matrix by reviewing the results of the Akaike Information Criterion (AIC) to evaluate goodness of fit for Model 2 and Model 3.

The sensitivity analysis investigated the association between MetS status and PA level by stratifying the main analyses according to the day of the week (weekday or weekend/holiday). We investigated the effect modification of the day of the week using the likelihood ratio test by comparing a multivariable model that included the interaction term between the day of the week and MetS with a multivariable model without this term.

All statistical analyses were performed using Stata/MP version 16.1 (Stata Corp LLC, College Station, TX, USA). A two-tailed *p*-value < 0.05 was considered statistically significant.

## 3. Results

### 3.1. Main Analyses

The 163 study participants (151 men and 12 women) included in the analyses had a median age (interquartile range [iqr]) of 44.2 [12.0] years. Of these, 31.8% of men (48/151) and 25.0% of women (3/12) were classified as having MetS. Table 1 shows the baseline characteristics according to MetS status. Median age [iqr] was highest in the MetS group (47.0 [10.0] years, *p* = 0.012). Although no significant differences were found, the MetS group had a high health awareness score (3.0 [3.0] points, *p* = 0.65), the highest proportion of participants indicating they considered the nutritional balance of their meals (70.6%, 36/51, *p* = 0.90), and the highest median step count (11,195 [5262] steps/day, *p* < 0.001) and active minutes (56 [60.2] mins/day, *p* < 0.001) among the groups.

Table 2 shows the association between MetS status and daily PA using Japanese PA reference values for the respective outcomes. Compared to the no-MetS group, the MetS group showed an inverse, albeit non-significant, association with step count in Model 1 (OR = 0.81; 95% CI: 0.47, 1.37). Following adjustment for additional covariates, the MetS group remained non-significantly and inversely associated with step count in Model 2 (OR = 0.86; CI: 0.50, 1.49) and Model 3 (OR = 0.81; 95% CI: 0.47, 1.40). Similarly, compared with the no-MetS group, the pre-MetS group showed an inverse, albeit non-significant, association with step count in Model 1 (OR = 0.63; 95% CI: 0.38, 1.04) and Model 2 (OR = 0.60; 95% CI: 0.36, 1.01). However, it showed a significant inverse association in the fully-adjusted model (Model 3: OR = 0.60; 95% CI: 0.36, 0.99).

In relation to active minutes, the MetS group showed a non-significant association in Model 1 (OR = 0.94; 95% CI: 0.57, 1.54), Model 2 (OR = 1.02; 95% CI: 0.63, 1.64), and Model 3 (OR = 0.94; 95% CI: 0.59, 1.49) compared to the no-MetS group. In contrast, the pre-MetS group showed an inverse, albeit non-significant, association with active minutes in Model 1 (OR = 0.63; 95% CI: 0.39, 1.01) compared to the no-MetS group. Following a further adjustment for covariates, however, the pre-MetS group showed a significant inverse association with active minutes in Model 2 (OR = 0.61; 95% CI: 0.39, 0.97) and Model 3 (OR = 0.62; 95% CI: 0.40, 0.96).

### 3.2. Sensitivity Analysis

Table 3 shows the main analyses stratified by the day of the week (i.e., weekday or weekend/holiday) for the association between MetS status and PA level. The analyses showed a significant difference in step count between weekdays and weekends/holidays in the MetS group (likelihood ratio test: *p* < 0.001). Compared to the weekday step count in the no-MetS group, MetS status was slightly positively, albeit non-significantly, associated with weekday step count in all models (Model 3: OR = 1.03; 95% CI: 0.59, 1.79). Meanwhile, compared to the weekday step count in the no-MetS group, pre-MetS status was inversely and non-significantly associated with weekday step count in Model 1 (OR = 0.61; 95% CI: 0.35, 1.06), but significantly and inversely associated with weekday step count in Model 2 (OR = 0.57; 95% CI: 0.33, 0.96) and Model 3 (OR = 0.57; 95% CI: 0.34, 0.95). When compared to the weekday step count in the no-MetS group, all MetS groups showed significantly inverse associations with weekend/holiday step count in all models (no-MetS Model 3: OR = 0.35; 95% CI: 0.27, 0.44; MetS Model 3: OR = 0.21; 95% CI: 0.12, 0.36; pre-MetS Model 3: OR = 0.23; 95% CI: 0.13, 0.38).

The analyses also showed a significant difference in active minutes between weekdays and weekends/holidays in the MetS group (likelihood ratio test: *p* < 0.001). Compared to the weekday active minutes in the no-MetS group, MetS status was slightly positively, albeit non-significantly, associated with weekday active minutes in all models (Model 3: OR = 1.09; 95% CI: 0.68, 1.75). Meanwhile, compared to the weekday active minutes in the no-MetS group, pre-MetS status was inversely and non-significantly associated with weekday active minutes in Model 1 (OR = 0.62; 95% CI: 0.38, 1.00), but inversely and significantly associated with weekday active minutes in Model 2 (OR = 0.60; 95% CI: 0.38, 0.96) and Model 3 (OR = 0.61; 95% CI: 0.39, 0.95). While no-MetS status was slightly positively albeit non-significantly associated with weekend/holiday active minutes in all models (Model 3: OR = 1.12; 95% CI: 0.91, 1.38), those in the MetS and pre-MetS groups showed a non-significant inverse association with weekend/holiday active minutes in all models (MetS Model 3: OR = 0.76; 95% CI: 0.47, 1.24; pre-MetS Model 3: OR = 0.71; 95% CI: 0.45, 1.12) compared to the weekday active minutes in the no-MetS group.

## 4. Discussion

### 4.1. Main Analysis

The present study aimed to examine the association between MetS status and PA level, as represented by step count and active minutes, using Japanese PA reference values recommended by EPAR. The main analysis indicated that the pre-MetS and MetS groups showed comparable associations with both step count and active minutes, respectively, when compared to the no-MetS group. Participants with MetS showed an inverse, albeit non-significant, association with both step count and active minutes compared to those with no MetS. Meanwhile, individuals with pre-MetS showed significant inverse associations with both step count and active minutes. In fact, compared to individuals without MetS, those with pre-MetS had approximately 40% lower odds of reaching their daily recommended step count and active minutes.

Our finding that MetS did not show a significant inverse association with PA deviates from the primary hypothesis. The following may explain these results. First, in Japan, insurers have been required to provide AHCs to insured employees aged 40 to 74 under the Japanese National Health Screening and Intervention Program since 2008. Employees classified as having MetS are requested to receive intensive health guidance from health professionals, which includes a follow-up consultation for 3–6 months [27], while those with a lower risk of developing lifestyle-related diseases are asked to undergo motivational health guidance with a professional’s advice. Some participants with MetS were likely aware of their health risk and engaged in positive health behaviors to improve their condition with active health support. In fact, we found that those with MetS, although not significant, had higher health awareness scores than the no-MetS group, more frequently indicated they intended to consume a balanced diet, and had higher median PA levels compared to the other MetS groups during the study period. Hence, participants with MetS may have comparatively higher health awareness [28]; in addition, the intervention itself may have re-affirmed health awareness and health-conscious behaviors in those with MetS in this study. To eliminate contingency and more precisely evaluate PA, a study of longer duration is needed in the future. Second, the study was conducted in winter, from December 2018 to March 2019. Many Japanese companies close for several days over the New Year’s holiday period, and some participants may adopt irregular lifestyle patterns or behaviors during this period. Our finding that PA levels varied among MetS individuals may be related to when the study was conducted, which may have affected the results. Characteristic seasonal behaviors [29] may thus need to be considered when researching PA. Third, while we used the reference values recommended by the EPAR to determine PA level (9000 steps/day in men, 8500 steps/day in women or 60 min MVPA/day, about 23 METs h/week), according to a suggestion by Kim et al. [30], an even higher PA level (26 METs h/week) may be more appropriate for the middle-aged Japanese population in order to reduce the risk of MetS. Finally, our participants were office workers in large enterprises with over 1000 employees, had relatively high incomes, and were predominantly men (92.6%). Because gender effects are often observed in the analysis of PA and MetS [30], the demographics and characteristics of this study population may have attenuated the associations between PA and MetS prevalence.

Conversely, pre-MetS, considered a transitional pathway to MetS, showed a significant inverse association with PA level, presenting approximately 40% lower odds of reaching the daily recommended PA level than the no-MetS group. This finding indicates that participants with pre-MetS had significantly lower PA levels than those with no MetS, and were unlikely to achieve the current PA guidelines. A notable difference between the MetS and the pre-MetS groups may be whether individuals are receiving active health support with follow-up consultation. As the pre-MetS group exhibited significantly lowest odds among the MetS groups, those with pre-MetS would benefit from increased guidance to prevent the onset of MetS. Furthermore, we also found a significant association between pre-MetS and PA level only after adjusting for health awareness in addition to lifestyle factors. This finding suggests that health awareness is an important covariate for the association between MetS and PA.

Given the non-significant associations between MetS and PA level, our results were inconsistent with previous studies that reported a clear inverse association between PA and MetS. However, it should be noted that the design of the present study differed from that of previous studies in that it examined the main exposure of MetS according to three categories and adjusted for a different selection of covariates. A study by Ko et al., which examined the association between PA and MetS in male white collar workers [31], reported that the prevalence of MetS was significantly lower (*p* < 0.05) in the high PA group (14.3%) than in the low PA group (25.2%), and that the latter had a higher risk of MetS than the former. Another study by Xu et al. [32] reported that high PA was significantly inversely associated with MetS after adjusting for age, sex, ethnicity, and current smoking in older adults with obesity. Importantly, these studies used questionnaires, namely the International Physical Activity Questionnaire (IPAQ) or Global Physical Activity Questionnaire (GPAQ), to assess the PA level, and a different index of MetS criteria. A systematic review and meta-analysis by Oliveira et al. [3] noted that studies of MetS in adolescents using self-reported assessments did not indicate a significant association, and suggested using objective measure instruments such as accelerometers. A study that objectively measured PA using a triaxial accelerometer and MetS index in Japan by Kim et al. [30] reported that low levels of PA were significantly positively associated with MetS compared with high levels of PA, with a significantly higher risk observed in middle-aged Japanese men, but not women. Similarly, Sagawa et al. [33] reported that a higher step count (≥10,000 steps/day) in middle-aged Japanese men objectively measured by a pedometer was inversely associated with MetS.

When measuring PA in free-living conditions, the use of wearable devices may avoid recall bias by participants. However, that proprietary algorithms are not disclosed leaves a level of uncertainty [17,24]. In fact, validation studies that compared wearable devices with gold standard instruments indicate that individual wearable devices are prone to variability in their measurement of specific PA parameters [18]. For example, findings of variability in MVPA estimations, which increase along with the MVPA volume [24,34], by Fitbit devices under free-living conditions compared to a gold standard have been attributed to the systematic bias related to differences in the algorithms of each device [24,35]. Although some limitations remain, the capabilities and accuracy of wearable devices for obtaining objective measures will likely increase with each software update and technological upgrade, which, when incorporated, should lead to their optimal usage in research.

Another explanation for our results could be that activity levels differ between weekdays and weekends/holidays. We conducted a sensitivity analysis with the hypothesis that the day of the week could be an effect modifier for the association between MetS status and PA level. Indeed, we found that the interaction between the day of the week and MetS status was highly significant for both step count and active minutes. Thus, the fact that the study period included several days over the New Year’s holidays could have contributed substantially if PA differed according to the day of the week.

### 4.2. Sensitivity Analysis

Our sensitivity analysis indicated that while all MetS groups had significantly lower step counts on weekends/holidays, active minutes did not significantly change in any group. Although the associations were not significant, participants with MetS were likely to meet the reference PA level on weekdays and have slightly higher PA than those with no MetS, but had decreased PA on weekends/holidays, with greater disparity observed by the day of the week in those with MetS. We speculate that most of the PA conducted by individuals with MetS may come from their weekday commute, leading to less activity on weekends/holidays. In fact, a study that examined the effect of sitting time on MetS in Japanese workers reported that a high proportion of sitting time in leisure time during holidays may increase the risk of MetS [36]. We also found a significant effect modification by the day of the week, with the step count of all MetS groups being lower on weekends/holidays than on weekdays. However, while the number of active minutes on weekends/holidays was lower than that on weekdays for those with MetS, it was unchanged for those with pre-MetS and higher for those with no MetS. Given that active minutes also provide information on the intensity of the PA, our results suggest that the modifying effect of the day of the week is greatest for those with MetS, who on weekends/holidays have a lower step count and fewer active minutes (i.e., less time spent in MVPA). In contrast, although those with no MetS had a lower step count on weekends/holidays, they spent more time in MVPA. We assume that those with no MetS and pre-MetS may conduct more intense PA, such as running, because of their lower step count on weekends/holidays. Our findings illustrate the important difference and characteristic pattern of PA among those with different MetS status by the day of the week according to current guidelines for PA.

Some studies have shown that those with MetS have lower energy expenditure in leisure time physical activity (LTPA) than those with no MetS [37], and that increased LTPA is significantly inversely associated with MetS [38]. In addition to leisure time, our findings suggest that the day of the week is another factor affecting the association between MetS and PA. Education to enhance health awareness and campaigns that promote PA on weekends/holidays might be effective public health strategies for preventing the onset of MetS. In particular, consistent PA regardless of the day of the week should be recommended for those with or at risk of MetS.

### 4.3. Limitations and Strengths

This study has a few limitations. First, as this was a cross-sectional analysis, we could not determine the causal associations between MetS and PA. Second, the cross-sectional analysis does not take into account the temporal change in activity for the 3 exposure groups during the 3-month study period. Third, the definition of pre-MetS and MetS relies on a combination of specific components (i.e., waist circumference, lipids, blood pressure, and blood glucose). Consequently, this may result in the heterogeneity of participants within the pre-MetS and MetS groups, respectively. However, the decomposition of the pre-MetS and MetS groups into component-specific classifications are beyond the scope of the present study. Moreover, the definitions of pre-MetS and MetS are in accord with Japanese national guidelines. Finally, the results of this study may not be generalizable to the Japanese general population given that the majority of participants were male office workers in Tokyo.

Despite these limitations, this study has several strengths. First, to our knowledge, this was the first analysis to use 3 MetS categories, namely no-MetS, pre-MetS and MetS, to investigate the association of MetS status with PA. Second, we examined real-world data objectively measured using a wearable device instead of relying on questionnaires to assess PA level. Third, this study also used anthropometric and biomarker data from AHCs. Finally, we investigated the impact of weekday/weekend PA level, which was made possible by using the wearable device.

## 5. Conclusions

Individuals with MetS did not show a significant inverse association with PA level compared to those with no MetS. In contrast, individuals with pre-MetS had significantly lower odds of reaching the step count and active minutes recommended by the Japanese Exercise and Physical Activity Reference for Health Promotion guidelines. Moreover, the day of the week (i.e., weekday vs weekend/holiday) was a modifier of the association between MetS status and PA level. We suggest that, in addition to those with MetS, those with pre-MetS would benefit from support strategies that encourage increased PA to reduce the risk of MetS onset. Continuous efforts to promote consistent PA including on weekends/holidays is also needed. Further research using longitudinal analyses with longer study periods, larger sample sizes, and numbers from both genders is needed to confirm our findings.

## Figures and Tables

**Table 1:** Baseline characteristics according to metabolic syndrome status (*n* = 163).

Characteristic	no-MetS *n* = 52	pre-MetS *n* = 60	MetS *n* = 51	*p* Value ^a^
Men, *n* (%)	43 (82.7)	60 (100.0)	48 (94.1)	0.002
Age at screening [years (median ± iqr)]	43.0 ± 10.0	43.5 ± 13.5	47.0 ±10.0	0.012
Weekday obs (%)	1670 (69.9)	2205 (68.8)	1825 (68.4)	0.52
Lifestyle factors				
Smoking status, *n* (%)				0.47
Non-smoker	28 (53.9)	24 (40.0)	22 (43.1)	
Past smoker	11 (21.2)	21 (35.0)	17 (33.3)	
Current smoker (<20 cigarettes/day)	5 (9.6)	10 (16.7)	6 (11.8)	
Current smoker (≥20 cigarettes/day)	8 (15.4)	5 (8.3)	6 (11.8)	
Living arrangement, *n* (%) Living with someone	47 (90.4)	54 (90.0)	46 (90.2)	1.00
Total hours of overtime/month [hours (median ± iqr)]	30 ± 27.5	42.5 ± 30.0	40 ± 32.0	0.24
Income, *n* (%)				0.42
<10 million JPY	37 (71.2)	39 (65.0)	30 (58.8)	
≥10 million JPY	15 (28.9)	21 (35.0)	21 (41.2)	
Diet (eating habit), *n* (%) Balanced food intake	36 (69.2)	40 (66.7)	36 (70.6)	0.90
Health awareness				
Health awareness score (7-point scale) [score (median ± iqr)]	2.5 ± 3.0	3.0 ± 2.0	3.0 ± 3.0	0.65
Health condition (5-point scale) [score (median ± iqr)]	3.0 ± 1.0	2.0 ± 1.0	2.0 ± 2.0	0.34
Daily components				
Number of steps/day [median ± iqr]	11,023 ± 5058	10,276 ± 5024	11,195 ± 5262	<0.001
Total active minutes/day [median ± iqr]	54 ± 53.6	48 ± 50.4	56 ± 60.2	<0.001
Total sleep time, hours/day [median ± iqr]	5.7 ± 1.4	5.6 ± 1.3	5.5 ± 1.3	0.001
Alcohol consumption, obs (%)				<0.001
<20 ethanol g/day	1323 (55.3)	1934 (60.3)	1640 (61.5)	
≥20 ethanol g/day	1068 (44.7)	1272 (39.7)	1028 (38.5)	

Abbreviations: obs: observations, MetS: metabolic syndrome, iqr: interquartile range. ^a^ Chi-squared test for categorical variables, Kruskal–Wallis test for continuous variables and daily components.

**Table 2:** Odds ratios of achieving the recommended physical activity level by metabolic syndrome status (*n* = 163).

Step Count		Model 1 ^a^	Model 2 ^b^	Model 3 ^c^
>9000/day in men and >8500/day in women		OR (95% CI)	OR (95% CI)	OR (95% CI)
	no-MetS	Reference	Reference	Reference
	pre-MetS	0.63 (0.38, 1.04)	0.60 (0.36, 1.01)	0.60 * (0.36, 0.99)
	MetS	0.81 (0.47, 1.37)	0.86 (0.50, 1.49)	0.81 (0.47, 1.40)
**Active minutes**		**Model 1 ^a^**	**Model 2 ^b^**	**Model 3 ^c^**
>3 METs h/day		OR (95% CI)	OR (95% CI)	OR (95% CI)
	no-MetS	Reference	Reference	Reference
	pre-MetS	0.63 (0.39, 1.01)	0.61 * (0.39, 0.97)	0.62 * (0.40, 0.96)
	MetS	0.94 (0.57, 1.54)	1.02 (0.63, 1.64)	0.94 (0.59, 1.49)

* *p* < 0.05. Abbreviations: MetS: metabolic syndrome, METs: Metabolic equivalent, OR: odds ratio, CI: confidence interval. Bold values denote statistically significant results compared with the no-MetS group. ^a^ Model 1 was adjusted for sex and age as fixed effects. ^b^ Model 2 was additionally adjusted for day of the week (weekday or weekend/holiday), balanced nutritional intake, smoking, overtime work, living arrangement, socio-economic status (all fixed effects), alcohol consumption, and total sleep time (random effects). ^c^ Model 3 was additionally adjusted for health awareness and self-rated health status.

**Table 3:** Odds ratios of achieving the recommended physical activity level by metabolic syndrome status on weekdays or weekends/holidays (*n* = 163).

Step Count		Model 1 ^a^	Model 2 ^b^	Model 3 ^c^
>9000/day in men and >8500/day in women		OR (95% CI)	OR (95% CI)	OR (95% CI)
Weekday	no-MetS	Reference	Reference	Reference
	pre-MetS	0.61 (0.35, 1.06)	0.57 * (0.33, 0.96)	0.57 * (0.34, 0.95)
	MetS	1.06 (0.59, 1.90)	1.09 (0.62, 1.92)	1.03 (0.59, 1.79)
weekend/holiday	no-MetS	0.32 ** (0.26, 0.40)	0.35 ** (0.27, 0.44)	0.35 ** (0.27, 0.44)
	pre-MetS	0.22 ** (0.12, 0.39)	0.23 ** (0.13, 0.39)	0.23 ** (0.13, 0.38)
	MetS	0.18 ** (0.10, 0.33)	0.22 ** (0.12, 0.39)	0.21 ** (0.12, 0.36)
**Active minutes**		**Model 1 ^a^**	**Model 2 ^b^**	**Model 3 ^c^**
>3 METs h/day		OR (95% CI)	OR (95% CI)	OR (95% CI)
weekday	no-MetS	Reference	Reference	Reference
weekend/holiday	pre-MetS	0.62 (0.38, 1.00)	0.60 * (0.38, 0.96)	0.61 * (0.39, 0.95)
	MetS	1.07 (0.64, 1.77)	1.19 (0.73, 1.94)	1.09 (0.68, 1.75)
	no-MetS	1.03 (0.85, 1.26)	1.12 (0.90, 1.38)	1.12 (0.91, 1.38)
	pre-MetS	0.66 (0.40, 1.09)	0.70 (0.43, 1.12)	0.71 (0.45, 1.12)
	MetS	0.72 (0.43, 1.21)	0.84 (0.51, 1.38)	0.76 (0.47, 1.24)

* *p* < 0.05, ** *p* < 0.01. Abbreviations: MetS: metabolic syndrome, METs: Metabolic equivalent, OR: odds ratio, CI: confidence interval. Bold values denote statistically significant results compared with the no-MetS group on weekday. ^a^ Model 1 was adjusted for sex and age as fixed effects. ^b^ Model 2 was additionally adjusted for balanced nutritional intake, smoking, overtime work, living arrangement, socio-economic status (all fixed effects), alcohol consumption, and total sleep time (random effects). ^c^ Model 3 was additionally adjusted for health awareness and self-rated health status.

## Data Availability

We cannot provide public access to individual data due to participant privacy stipulations in accordance with ethical guidelines. Additionally, the written informed consent we obtained from study participants does not include a provision for publicly sharing data. Qualifying researchers may, upon reasonable request, apply to access an aggregated dataset by contacting the corresponding author.
